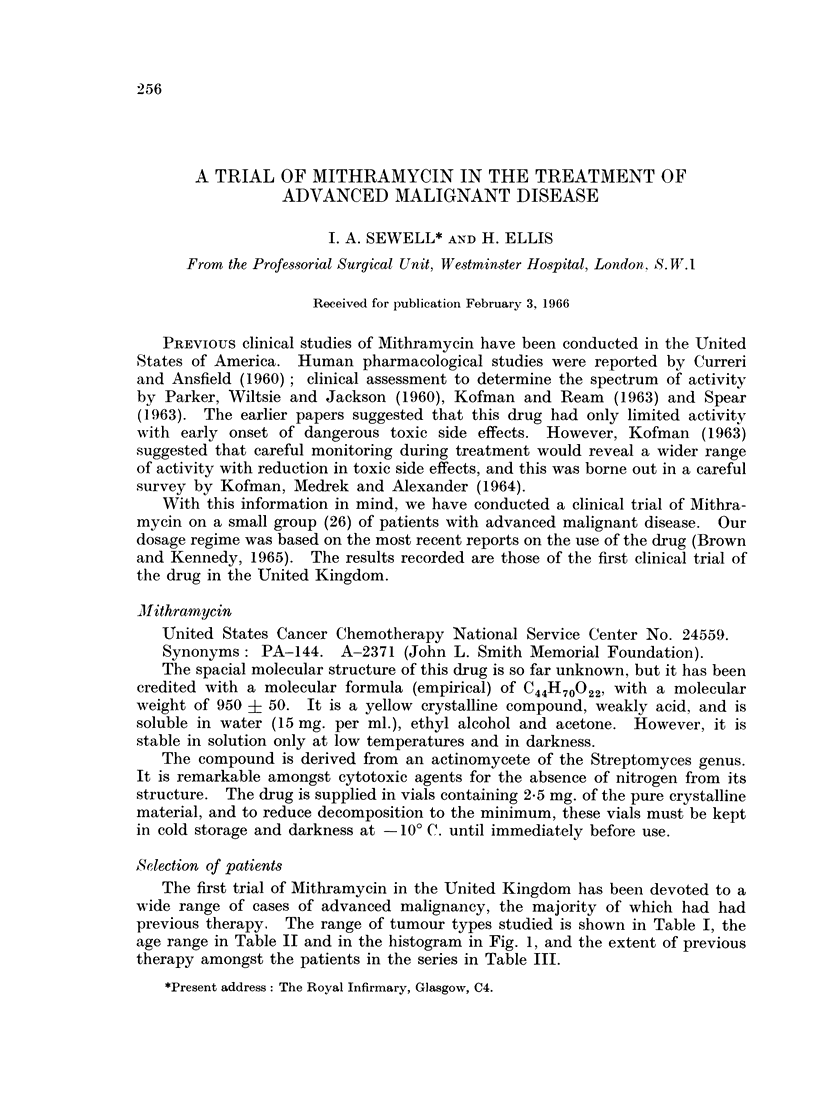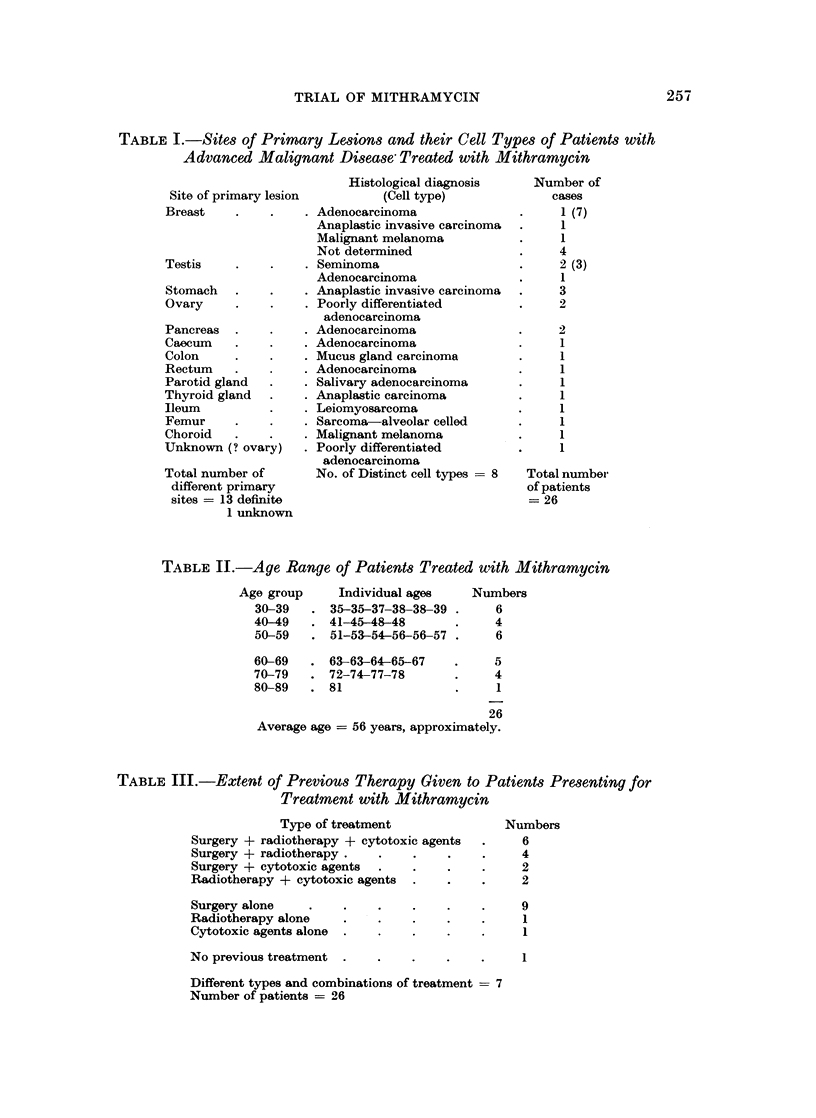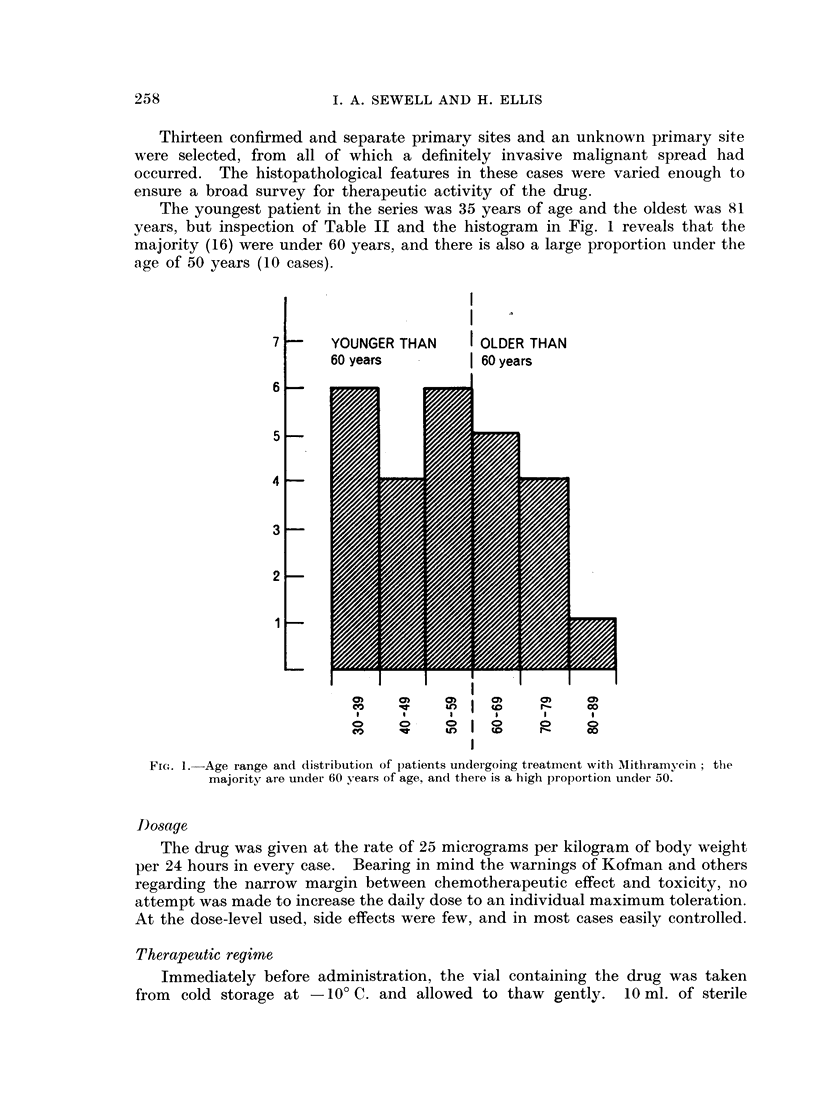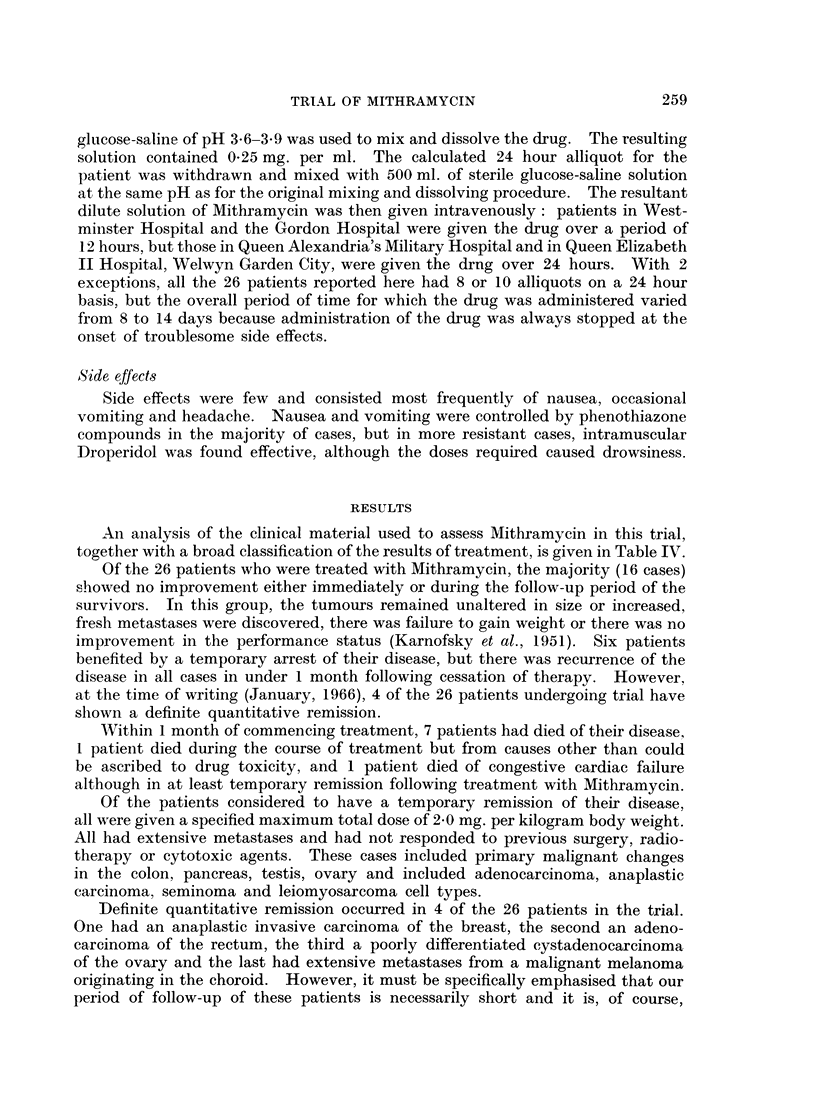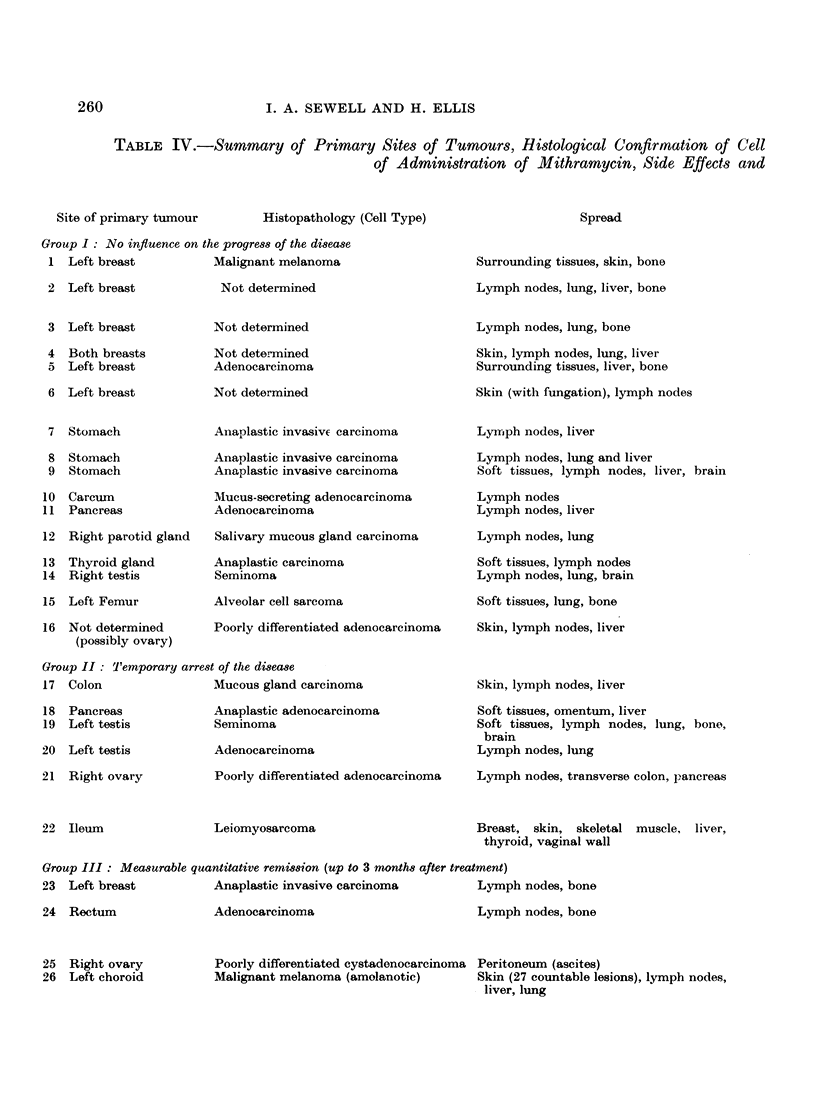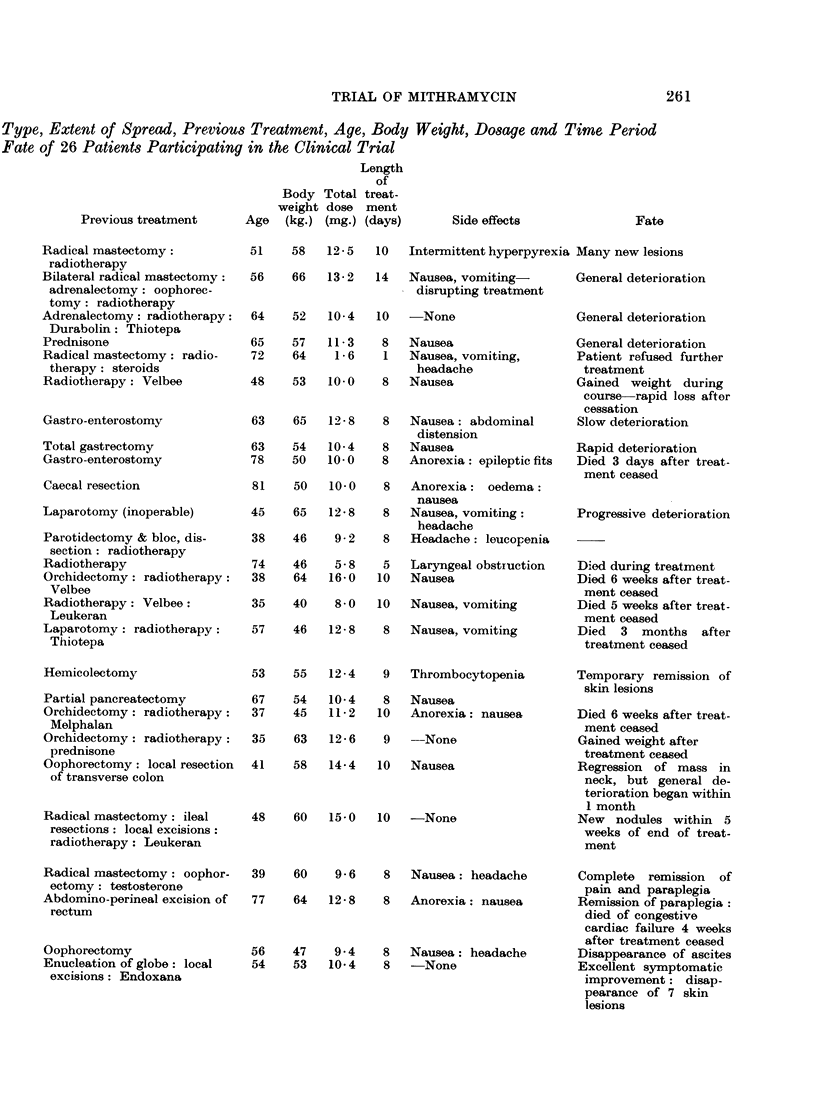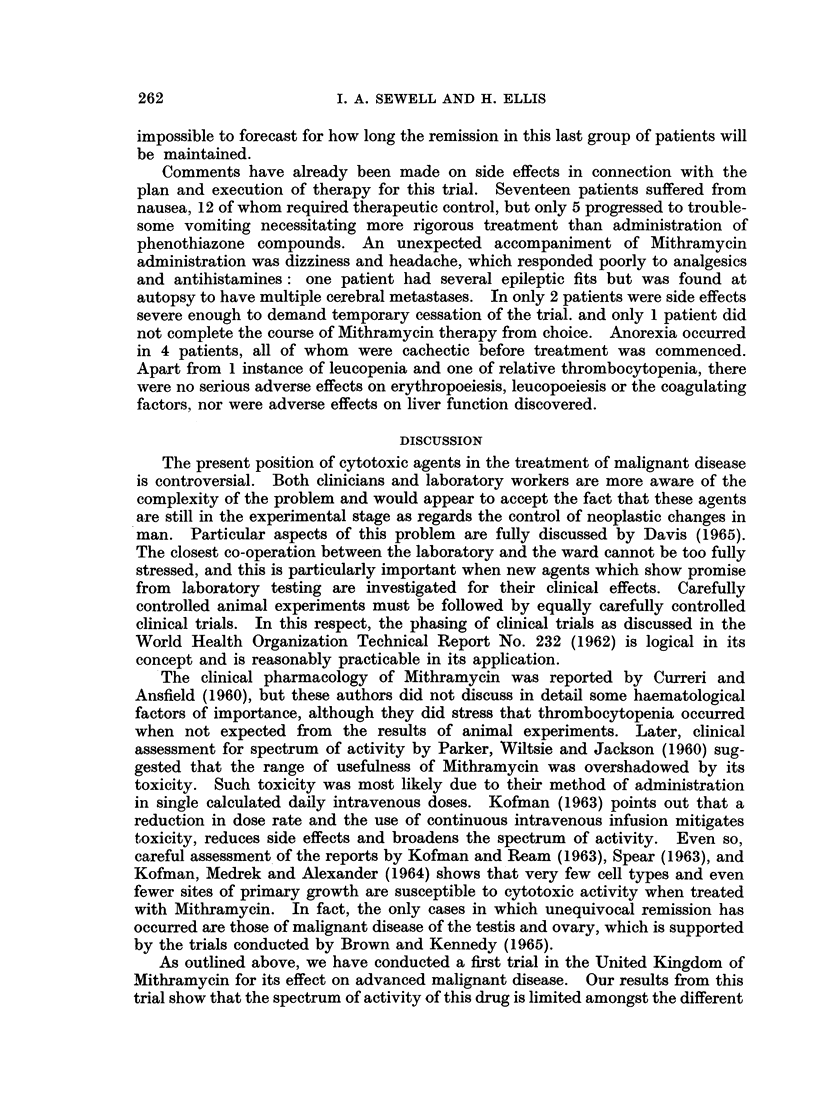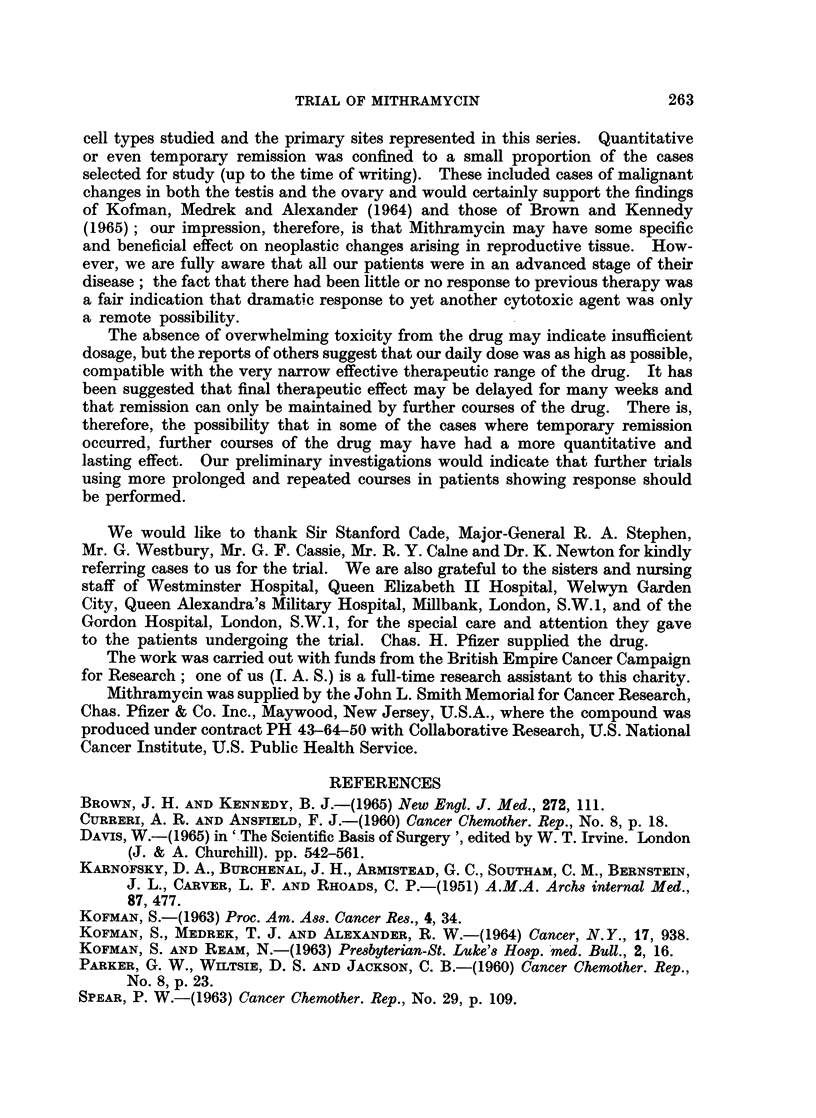# A trial of mithramycin in the treatment of advanced malignant disease.

**DOI:** 10.1038/bjc.1966.32

**Published:** 1966-06

**Authors:** I. A. Sewell, H. Ellis


### A TRIAL OF MITHRAMYCIN IN THE TREATMENT OF ADVANCED MALIGNANT DISEASE

I. A. SEWELL* AND H. ELLIS

*From the Professorial Surgical Unit, Westminster Hospital, London, S.W.1*

Received for publication February 3, 1966

PREVIOUS clinical studies of Mithramycin have been conducted in the United States of America. Human pharmacological studies were reported by Curreri and Ansfield (1960) ; clinical assessment to determine the spectrum of activity by Parker, Wiltsie and Jackson (1960), Kofman and Ream (1963) and Spear (1963). The earlier papers suggested that this drug had only limited activity with early onset of dangerous toxic side effects. However, Kofman (1963) suggested that careful monitoring during treatment would reveal a wider range of activity with reduction in toxic side effects, and this was borne out in a careful survey by Kofman, Medrek and Alexander (1964).

With this information in mind, we have conducted a clinical trial of Mithramycin on a small group (26) of patients with advanced malignant disease. Our dosage regime was based on the most recent reports on the use of the drug (Brown and Kennedy, 1965). The results recorded are those of the first clinical trial of the drug in the United Kingdom.

### *Mithramycin*

United States Cancer Chemotherapy National Service Center No. 24559.

Synonyms : PA–144. A–2371 (John L. Smith Memorial Foundation).

The spacial molecular structure of this drug is so far unknown, but it has been credited with a molecular formula (empirical) of $C_{44}H_{70}O_{22}$, with a molecular weight of $950 \pm 50$. It is a yellow crystalline compound, weakly acid, and is soluble in water (15 mg. per ml.), ethyl alcohol and acetone. However, it is stable in solution only at low temperatures and in darkness.

The compound is derived from an actinomycete of the Streptomyces genus. It is remarkable amongst cytotoxic agents for the absence of nitrogen from its structure. The drug is supplied in vials containing 2·5 mg. of the pure crystalline material, and to reduce decomposition to the minimum, these vials must be kept in cold storage and darkness at $-10°$ C. until immediately before use.

### *Selection of patients*

The first trial of Mithramycin in the United Kingdom has been devoted to a wide range of cases of advanced malignancy, the majority of which had had previous therapy. The range of tumour types studied is shown in Table I, the age range in Table II and in the histogram in Fig. 1, and the extent of previous therapy amongst the patients in the series in Table III.

*Present address : The Royal Infirmary, Glasgow, C4.

Table I.—*Sites of Primary Lesions and their Cell Types of Patients with Advanced Malignant Disease Treated with Mithramycin*

| Site of primary lesion | Histological diagnosis (Cell type) | Number of cases |
|---|---|---|
| Breast | Adenocarcinoma | 1 (7) |
| | Anaplastic invasive carcinoma | 1 |
| | Malignant melanoma | 1 |
| | Not determined | 4 |
| Testis | Seminoma | 2 (3) |
| | Adenocarcinoma | 1 |
| Stomach | Anaplastic invasive carcinoma | 3 |
| Ovary | Poorly differentiated adenocarcinoma | 2 |
| Pancreas | Adenocarcinoma | 2 |
| Caecum | Adenocarcinoma | 1 |
| Colon | Mucus gland carcinoma | 1 |
| Rectum | Adenocarcinoma | 1 |
| Parotid gland | Salivary adenocarcinoma | 1 |
| Thyroid gland | Anaplastic carcinoma | 1 |
| Ileum | Leiomyosarcoma | 1 |
| Femur | Sarcoma—alveolar celled | 1 |
| Choroid | Malignant melanoma | 1 |
| Unknown (? ovary) | Poorly differentiated adenocarcinoma | 1 |
| Total number of different primary sites = 13 definite, 1 unknown | No. of Distinct cell types = 8 | Total number of patients = 26 |

Table II.—*Age Range of Patients Treated with Mithramycin*

| Age group | Individual ages | Numbers |
|---|---|---|
| 30–39 | 35–35–37–38–38–39 | 6 |
| 40–49 | 41–45–48–48 | 4 |
| 50–59 | 51–53–54–56–56–57 | 6 |
| 60–69 | 63–63–64–65–67 | 5 |
| 70–79 | 72–74–77–78 | 4 |
| 80–89 | 81 | 1 |
| | | 26 |

Average age = 56 years, approximately.

Table III.—*Extent of Previous Therapy Given to Patients Presenting for Treatment with Mithramycin*

| Type of treatment | Numbers |
|---|---|
| Surgery + radiotherapy + cytotoxic agents | 6 |
| Surgery + radiotherapy | 4 |
| Surgery + cytotoxic agents | 2 |
| Radiotherapy + cytotoxic agents | 2 |
| Surgery alone | 9 |
| Radiotherapy alone | 1 |
| Cytotoxic agents alone | 1 |
| No previous treatment | 1 |

Different types and combinations of treatment = 7
Number of patients = 26

Thirteen confirmed and separate primary sites and an unknown primary site were selected, from all of which a definitely invasive malignant spread had occurred. The histopathological features in these cases were varied enough to ensure a broad survey for therapeutic activity of the drug.

The youngest patient in the series was 35 years of age and the oldest was 81 years, but inspection of Table II and the histogram in Fig. 1 reveals that the majority (16) were under 60 years, and there is also a large proportion under the age of 50 years (10 cases).

FIG. 1.—Age range and distribution of patients undergoing treatment with Mithramycin ; the majority are under 60 years of age, and there is a high proportion under 50.

*Dosage*

The drug was given at the rate of 25 micrograms per kilogram of body weight per 24 hours in every case. Bearing in mind the warnings of Kofman and others regarding the narrow margin between chemotherapeutic effect and toxicity, no attempt was made to increase the daily dose to an individual maximum toleration. At the dose-level used, side effects were few, and in most cases easily controlled.

*Therapeutic regime*

Immediately before administration, the vial containing the drug was taken from cold storage at $-10°$ C. and allowed to thaw gently. 10 ml. of sterile glucose-saline of pH 3·6–3·9 was used to mix and dissolve the drug. The resulting solution contained 0·25 mg. per ml. The calculated 24 hour alliquot for the patient was withdrawn and mixed with 500 ml. of sterile glucose-saline solution at the same pH as for the original mixing and dissolving procedure. The resultant dilute solution of Mithramycin was then given intravenously : patients in Westminster Hospital and the Gordon Hospital were given the drug over a period of 12 hours, but those in Queen Alexandria's Military Hospital and in Queen Elizabeth II Hospital, Welwyn Garden City, were given the drng over 24 hours. With 2 exceptions, all the 26 patients reported here had 8 or 10 alliquots on a 24 hour basis, but the overall period of time for which the drug was administered varied from 8 to 14 days because administration of the drug was always stopped at the onset of troublesome side effects.

*Side effects*

Side effects were few and consisted most frequently of nausea, occasional vomiting and headache. Nausea and vomiting were controlled by phenothiazone compounds in the majority of cases, but in more resistant cases, intramuscular Droperidol was found effective, although the doses required caused drowsiness.

### RESULTS

An analysis of the clinical material used to assess Mithramycin in this trial, together with a broad classification of the results of treatment, is given in Table IV.

Of the 26 patients who were treated with Mithramycin, the majority (16 cases) showed no improvement either immediately or during the follow-up period of the survivors. In this group, the tumours remained unaltered in size or increased, fresh metastases were discovered, there was failure to gain weight or there was no improvement in the performance status (Karnofsky *et al.*, 1951). Six patients benefited by a temporary arrest of their disease, but there was recurrence of the disease in all cases in under 1 month following cessation of therapy. However, at the time of writing (January, 1966), 4 of the 26 patients undergoing trial have shown a definite quantitative remission.

Within 1 month of commencing treatment, 7 patients had died of their disease, 1 patient died during the course of treatment but from causes other than could be ascribed to drug toxicity, and 1 patient died of congestive cardiac failure although in at least temporary remission following treatment with Mithramycin.

Of the patients considered to have a temporary remission of their disease, all were given a specified maximum total dose of 2·0 mg. per kilogram body weight. All had extensive metastases and had not responded to previous surgery, radiotherapy or cytotoxic agents. These cases included primary malignant changes in the colon, pancreas, testis, ovary and included adenocarcinoma, anaplastic carcinoma, seminoma and leiomyosarcoma cell types.

Definite quantitative remission occurred in 4 of the 26 patients in the trial. One had an anaplastic invasive carcinoma of the breast, the second an adenocarcinoma of the rectum, the third a poorly differentiated cystadenocarcinoma of the ovary and the last had extensive metastases from a malignant melanoma originating in the choroid. However, it must be specifically emphasised that our period of follow-up of these patients is necessarily short and it is, of course,

TABLE IV.—*Summary of Primary Sites of Tumours, Histological Confirmation of Cell of Administration of Mithramycin, Side Effects and*

| | Site of primary tumour | Histopathology (Cell Type) | Spread |
|---|---|---|---|
| *Group I : No influence on the progress of the disease* | | | |
| 1 | Left breast | Malignant melanoma | Surrounding tissues, skin, bone |
| 2 | Left breast | Not determined | Lymph nodes, lung, liver, bone |
| 3 | Left breast | Not determined | Lymph nodes, lung, bone |
| 4 | Both breasts | Not determined | Skin, lymph nodes, lung, liver |
| 5 | Left breast | Adenocarcinoma | Surrounding tissues, liver, bone |
| 6 | Left breast | Not determined | Skin (with fungation), lymph nodes |
| 7 | Stomach | Anaplastic invasive carcinoma | Lymph nodes, liver |
| 8 | Stomach | Anaplastic invasive carcinoma | Lymph nodes, lung and liver |
| 9 | Stomach | Anaplastic invasive carcinoma | Soft tissues, lymph nodes, liver, brain |
| 10 | Carcum | Mucus-secreting adenocarcinoma | Lymph nodes |
| 11 | Pancreas | Adenocarcinoma | Lymph nodes, liver |
| 12 | Right parotid gland | Salivary mucous gland carcinoma | Lymph nodes, lung |
| 13 | Thyroid gland | Anaplastic carcinoma | Soft tissues, lymph nodes |
| 14 | Right testis | Seminoma | Lymph nodes, lung, brain |
| 15 | Left Femur | Alveolar cell sarcoma | Soft tissues, lung, bone |
| 16 | Not determined (possibly ovary) | Poorly differentiated adenocarcinoma | Skin, lymph nodes, liver |
| *Group II : Temporary arrest of the disease* | | | |
| 17 | Colon | Mucous gland carcinoma | Skin, lymph nodes, liver |
| 18 | Pancreas | Anaplastic adenocarcinoma | Soft tissues, omentum, liver |
| 19 | Left testis | Seminoma | Soft tissues, lymph nodes, lung, bone, brain |
| 20 | Left testis | Adenocarcinoma | Lymph nodes, lung |
| 21 | Right ovary | Poorly differentiated adenocarcinoma | Lymph nodes, transverse colon, pancreas |
| 22 | Ileum | Leiomyosarcoma | Breast, skin, skeletal muscle, liver, thyroid, vaginal wall |
| *Group III : Measurable quantitative remission (up to 3 months after treatment)* | | | |
| 23 | Left breast | Anaplastic invasive carcinoma | Lymph nodes, bone |
| 24 | Rectum | Adenocarcinoma | Lymph nodes, bone |
| 25 | Right ovary | Poorly differentiated cystadenocarcinoma | Peritoneum (ascites) |
| 26 | Left choroid | Malignant melanoma (amolanotic) | Skin (27 countable lesions), lymph nodes, liver, lung |

*Type, Extent of Spread, Previous Treatment, Age, Body Weight, Dosage and Time Period*
*Fate of 26 Patients Participating in the Clinical Trial*

| Previous treatment | Age | Body weight (kg.) | Total dose (mg.) | Length of treatment (days) | Side effects | Fate |
|---|---|---|---|---|---|---|
| Radical mastectomy : radiotherapy | 51 | 58 | 12·5 | 10 | Intermittent hyperpyrexia | Many new lesions |
| Bilateral radical mastectomy : adrenalectomy : oophorectomy : radiotherapy | 56 | 66 | 13·2 | 14 | Nausea, vomiting—disrupting treatment | General deterioration |
| Adrenalectomy : radiotherapy : Durabolin : Thiotepa | 64 | 52 | 10·4 | 10 | —None | General deterioration |
| Prednisone | 65 | 57 | 11·3 | 8 | Nausea | General deterioration |
| Radical mastectomy : radiotherapy : steroids | 72 | 64 | 1·6 | 1 | Nausea, vomiting, headache | Patient refused further treatment |
| Radiotherapy : Velbee | 48 | 53 | 10·0 | 8 | Nausea | Gained weight during course—rapid loss after cessation |
| Gastro-enterostomy | 63 | 65 | 12·8 | 8 | Nausea : abdominal distension | Slow deterioration |
| Total gastrectomy | 63 | 54 | 10·4 | 8 | Nausea | Rapid deterioration |
| Gastro-enterostomy | 78 | 50 | 10·0 | 8 | Anorexia : epileptic fits | Died 3 days after treatment ceased |
| Caecal resection | 81 | 50 | 10·0 | 8 | Anorexia : oedema : nausea | |
| Laparotomy (inoperable) | 45 | 65 | 12·8 | 8 | Nausea, vomiting : headache | Progressive deterioration |
| Parotidectomy & bloc, dissection : radiotherapy | 38 | 46 | 9·2 | 8 | Headache : leucopenia | —— |
| Radiotherapy | 74 | 46 | 5·8 | 5 | Laryngeal obstruction | Died during treatment |
| Orchidectomy : radiotherapy : Velbee | 38 | 64 | 16·0 | 10 | Nausea | Died 6 weeks after treatment ceased |
| Radiotherapy : Velbee : Leukeran | 35 | 40 | 8·0 | 10 | Nausea, vomiting | Died 5 weeks after treatment ceased |
| Laparotomy : radiotherapy : Thiotepa | 57 | 46 | 12·8 | 8 | Nausea, vomiting | Died 3 months after treatment ceased |
| Hemicolectomy | 53 | 55 | 12·4 | 9 | Thrombocytopenia | Temporary remission of skin lesions |
| Partial pancreatectomy | 67 | 54 | 10·4 | 8 | Nausea | |
| Orchidectomy : radiotherapy : Melphalan | 37 | 45 | 11·2 | 10 | Anorexia : nausea | Died 6 weeks after treatment ceased |
| Orchidectomy : radiotherapy : prednisone | 35 | 63 | 12·6 | 9 | —None | Gained weight after treatment ceased |
| Oophorectomy : local resection of transverse colon | 41 | 58 | 14·4 | 10 | Nausea | Regression of mass in neck, but general deterioration began within 1 month |
| Radical mastectomy : ileal resections : local excisions : radiotherapy : Leukeran | 48 | 60 | 15·0 | 10 | —None | New nodules within 5 weeks of end of treatment |
| Radical mastectomy : oophorectomy : testosterone | 39 | 60 | 9·6 | 8 | Nausea : headache | Complete remission of pain and paraplegia |
| Abdomino-perineal excision of rectum | 77 | 64 | 12·8 | 8 | Anorexia : nausea | Remission of paraplegia : died of congestive cardiac failure 4 weeks after treatment ceased |
| Oophorectomy | 56 | 47 | 9·4 | 8 | Nausea : headache | Disappearance of ascites |
| Enucleation of globe : local excisions : Endoxana | 54 | 53 | 10·4 | 8 | —None | Excellent symptomatic improvement : disappearance of 7 skin lesions |

impossible to forecast for how long the remission in this last group of patients will be maintained.

Comments have already been made on side effects in connection with the plan and execution of therapy for this trial. Seventeen patients suffered from nausea, 12 of whom required therapeutic control, but only 5 progressed to troublesome vomiting necessitating more rigorous treatment than administration of phenothiazone compounds. An unexpected accompaniment of Mithramycin administration was dizziness and headache, which responded poorly to analgesics and antihistamines : one patient had several epileptic fits but was found at autopsy to have multiple cerebral metastases. In only 2 patients were side effects severe enough to demand temporary cessation of the trial. and only 1 patient did not complete the course of Mithramycin therapy from choice. Anorexia occurred in 4 patients, all of whom were cachectic before treatment was commenced. Apart from 1 instance of leucopenia and one of relative thrombocytopenia, there were no serious adverse effects on erythropoeiesis, leucopoeiesis or the coagulating factors, nor were adverse effects on liver function discovered.

### DISCUSSION

The present position of cytotoxic agents in the treatment of malignant disease is controversial. Both clinicians and laboratory workers are more aware of the complexity of the problem and would appear to accept the fact that these agents are still in the experimental stage as regards the control of neoplastic changes in man. Particular aspects of this problem are fully discussed by Davis (1965). The closest co-operation between the laboratory and the ward cannot be too fully stressed, and this is particularly important when new agents which show promise from laboratory testing are investigated for their clinical effects. Carefully controlled animal experiments must be followed by equally carefully controlled clinical trials. In this respect, the phasing of clinical trials as discussed in the World Health Organization Technical Report No. 232 (1962) is logical in its concept and is reasonably practicable in its application.

The clinical pharmacology of Mithramycin was reported by Curreri and Ansfield (1960), but these authors did not discuss in detail some haematological factors of importance, although they did stress that thrombocytopenia occurred when not expected from the results of animal experiments. Later, clinical assessment for spectrum of activity by Parker, Wiltsie and Jackson (1960) suggested that the range of usefulness of Mithramycin was overshadowed by its toxicity. Such toxicity was most likely due to their method of administration in single calculated daily intravenous doses. Kofman (1963) points out that a reduction in dose rate and the use of continuous intravenous infusion mitigates toxicity, reduces side effects and broadens the spectrum of activity. Even so, careful assessment of the reports by Kofman and Ream (1963), Spear (1963), and Kofman, Medrek and Alexander (1964) shows that very few cell types and even fewer sites of primary growth are susceptible to cytotoxic activity when treated with Mithramycin. In fact, the only cases in which unequivocal remission has occurred are those of malignant disease of the testis and ovary, which is supported by the trials conducted by Brown and Kennedy (1965).

As outlined above, we have conducted a first trial in the United Kingdom of Mithramycin for its effect on advanced malignant disease. Our results from this trial show that the spectrum of activity of this drug is limited amongst the different cell types studied and the primary sites represented in this series. Quantitative or even temporary remission was confined to a small proportion of the cases selected for study (up to the time of writing). These included cases of malignant changes in both the testis and the ovary and would certainly support the findings of Kofman, Medrek and Alexander (1964) and those of Brown and Kennedy (1965); our impression, therefore, is that Mithramycin may have some specific and beneficial effect on neoplastic changes arising in reproductive tissue. However, we are fully aware that all our patients were in an advanced stage of their disease; the fact that there had been little or no response to previous therapy was a fair indication that dramatic response to yet another cytotoxic agent was only a remote possibility.

The absence of overwhelming toxicity from the drug may indicate insufficient dosage, but the reports of others suggest that our daily dose was as high as possible, compatible with the very narrow effective therapeutic range of the drug. It has been suggested that final therapeutic effect may be delayed for many weeks and that remission can only be maintained by further courses of the drug. There is, therefore, the possibility that in some of the cases where temporary remission occurred, further courses of the drug may have had a more quantitative and lasting effect. Our preliminary investigations would indicate that further trials using more prolonged and repeated courses in patients showing response should be performed.

We would like to thank Sir Stanford Cade, Major-General R. A. Stephen, Mr. G. Westbury, Mr. G. F. Cassie, Mr. R. Y. Calne and Dr. K. Newton for kindly referring cases to us for the trial. We are also grateful to the sisters and nursing staff of Westminster Hospital, Queen Elizabeth II Hospital, Welwyn Garden City, Queen Alexandra's Military Hospital, Millbank, London, S.W.1, and of the Gordon Hospital, London, S.W.1, for the special care and attention they gave to the patients undergoing the trial. Chas. H. Pfizer supplied the drug.

The work was carried out with funds from the British Empire Cancer Campaign for Research; one of us (I. A. S.) is a full-time research assistant to this charity.

Mithramycin was supplied by the John L. Smith Memorial for Cancer Research, Chas. Pfizer & Co. Inc., Maywood, New Jersey, U.S.A., where the compound was produced under contract PH 43–64–50 with Collaborative Research, U.S. National Cancer Institute, U.S. Public Health Service.

### REFERENCES

BROWN, J. H. AND KENNEDY, B. J.—(1965) *New Engl. J. Med.*, **272**, 111.

CURRERI, A. R. AND ANSFIELD, F. J.—(1960) *Cancer Chemother. Rep.*, No. 8, p. 18.

DAVIS, W.—(1965) in 'The Scientific Basis of Surgery', edited by W. T. Irvine. London (J. & A. Churchill). pp. 542–561.

KARNOFSKY, D. A., BURCHENAL, J. H., ARMISTEAD, G. C., SOUTHAM, C. M., BERNSTEIN, J. L., CARVER, L. F. AND RHOADS, C. P.—(1951) *A.M.A. Archs internal Med.*, **87**, 477.

KOFMAN, S.—(1963) *Proc. Am. Ass. Cancer Res.*, **4**, 34.

KOFMAN, S., MEDREK, T. J. AND ALEXANDER, R. W.—(1964) *Cancer, N.Y.*, **17**, 938.

KOFMAN, S. AND REAM, N.—(1963) *Presbyterian-St. Luke's Hosp. med. Bull.*, **2**, 16.

PARKER, G. W., WILTSIE, D. S. AND JACKSON, C. B.—(1960) *Cancer Chemother. Rep.*, No. 8, p. 23.

SPEAR, P. W.—(1963) *Cancer Chemother. Rep.*, No. 29, p. 109.